# Habituation to Experimentally Induced Electrical Pain during Voluntary-Breathing Controlled Electrical Stimulation (BreEStim)

**DOI:** 10.1371/journal.pone.0104729

**Published:** 2014-08-25

**Authors:** Shengai Li, Tracy Hu, Maria A. Beran, Sheng Li

**Affiliations:** 1 Department of Physical Medicine and Rehabilitation, University of Texas Health Science Center at Houston, Houston, Texas, United States of America; 2 Neurorehabilitation Research Laboratory TIRR Memorial Hermann Research Center, Houston, Texas, United States of America; Duke University, United States of America

## Abstract

**Objective:**

Painful peripheral electrical stimulation to acupuncture points was found to cause sensitization if delivered randomly (EStim), but induced habituation if triggered by voluntary breathing (BreEStim). The objective was to systematically compare the effectiveness of BreEStim and EStim and to investigate the possible mechanisms mediating the habituation effect of BreEStim.

**Methods:**

Eleven pain-free, healthy subjects (6 males, 5 females) participated in the study. Each subject received the BreEStim and EStim treatments in a random order at least three days apart. Both treatments consisted of 120 painful but tolerable stimuli to the ulnar nerve at the elbow on the dominant arm. BreEStim was triggered by voluntary breathing while EStim was delivered randomly. Electrical sensation threshold (EST) and electrical pain threshold (EPT) were measured from the thenar and hypothenar eminences on both hands at pre-intervention and 10-minutes post-intervention.

**Results:**

There was no difference in the pre-intervention baseline measurement of EST and EPT between BreEStim and EStim. BreEStim increased EPT in all tested sites on both hands, while EStim increased EPT in the dominant hypothenar eminence distal to the stimulating site and had no effect on EPT in other sites. There was no difference in the intensity of electrical stimulation between EStim and BreEStim.

**Conclusion:**

Our findings support the important role human voluntary breathing plays in the systemic habituation effect of BreEStim to peripheral painful electrical stimulation.

## Introduction

Pain is multi-dimensional and includes distinct sensory and affective (i.e., unpleasantness) components [1]. Memory mechanisms play a significant role in the persistent awareness of chronic neuropathic pain as well as in the reinforcement of the associated distress. Neuropathic pain is very common, difficult to manage, and has increasingly been recognized as a major contributor to suffering, poor rehabilitation outcomes and reduced quality of life of the persons who are suffering from chronic neuropathic pain [2]–[4]. Various neurostimulation techniques [5], such as transcutaneous electrical nerve stimulation (TENS) [6], electroacupuncture [7], spinal cord stimulation [8], deep brain stimulation [9], and transcranial direct current stimulation [10]–[13] have been used for management of neuropathic pain.

We recently proposed an innovative treatment – breathing-controlled electrical stimulation (BreEStim) for neuropathic pain management [14], [15]. This technique was developed from our discovery of the systemic effect of human voluntary breathing on motor function and pain perception [14]–[20]. In the BreEStim treatment (see details in [14]), human voluntary breathing signal triggers an external electrical stimulator. A single-pulse electrical stimulation is then delivered to peripheral acupuncture points. After receiving a week of daily BreEStim treatment to the acupuncture points on the ipsilateral forearm, a patient with constant shooting phantom pain secondary to an above-the-knee amputation reported no more shooting phantom pain, although he was still able to feel the occasional non-painful shooting sensation in the phantom limb [15]. A similar analgesic effect was also reported in a patient with spinal cord injury [14]. To account for this analgesic effect, we hypothesized that BreEStim integrates multiple internal pain coping mechanisms during voluntary breathing [15], such as electroacupuncture effect, habituation to aversive stimuli, analgesia effect from voluntary breathing, anterograde amnesia of aversive stimuli and activation of the reward system. As a result, the BreEStim treatment has a measurable clinical analgesic effect by increasing pain tolerance, i.e., increased pain threshold.

In a recent study [21], we compared the effects of BreEStim and EStim on sensory thresholds in healthy subjects. Electrical pain threshold (EPT) increased after BreEStim, but decreased after EStim. Neither intervention affected other sensory thresholds (mechanical sensation threshold, thermal thresholds). In this study [21], the same protocol (100 painful electrical stimuli at similar intensities delivered to acupuncture points, Neiguan and Weiguan, on the forearm) led to the opposite findings of habituation after BreEStim and sensitization after EStim. This protocol, however, had - a few confounding factors. The location of the acupuncture point (Neiguan) is very close to the path of the median nerve. Thus, electrical stimulation to the acupuncture points would stimulate the median nerve as well. Both repeated aversive electrical stimulation and electro-acupuncture are reported to have analgesic effects [7], [22], [23], but the sensitization effect was only observed after EStim. Following unilateral electrical median nerve stimulation, there was bilateral activation of primary somatosensory cortex [22] and increased pain threshold of the contralateral index finger [23]. In contrast, the sensitization effect was seen in both stimulated and contralateral symmetrical area after EStim in the previous study [21]. In the present study, we aimed to compare BreEStim and EStim by examining these factors (nerve stimulation vs. acupuncture, unilateral vs. contralateral or systemic effects).

## Methods

### Subjects

Eleven young and healthy subjects (6 male, 5 female, averaged 29.7 years of age, ranging from 25–44) volunteered in this experiment. According to daily use in writing and eating, one subject was left-handed, and the rest were right-headed. All subjects had no known history of neuromuscular diseases and were pain free. All subjects gave written informed consent prior to participation. This study was approved by the Committee for the Protection of Human Subjects at the University of Texas Health Science Center at Houston and TIRR Memorial Hermann Hospital.

### Experimental procedures and BreEStim/EStim interventions

In the present study, we adopted our recently published protocol [21]. Each subject received two intervention sessions – EStim and BreEStim. Each intervention was given at least 3 days apart and the order of the sessions was randomized and balanced across subjects to minimize the order effect. Each intervention session consisted of 120 stimuli and took about 30–40 min. Details of each intervention are available on the open access methodology video article at: http://www.jove.com/video/50077/.

**Figure 1:**
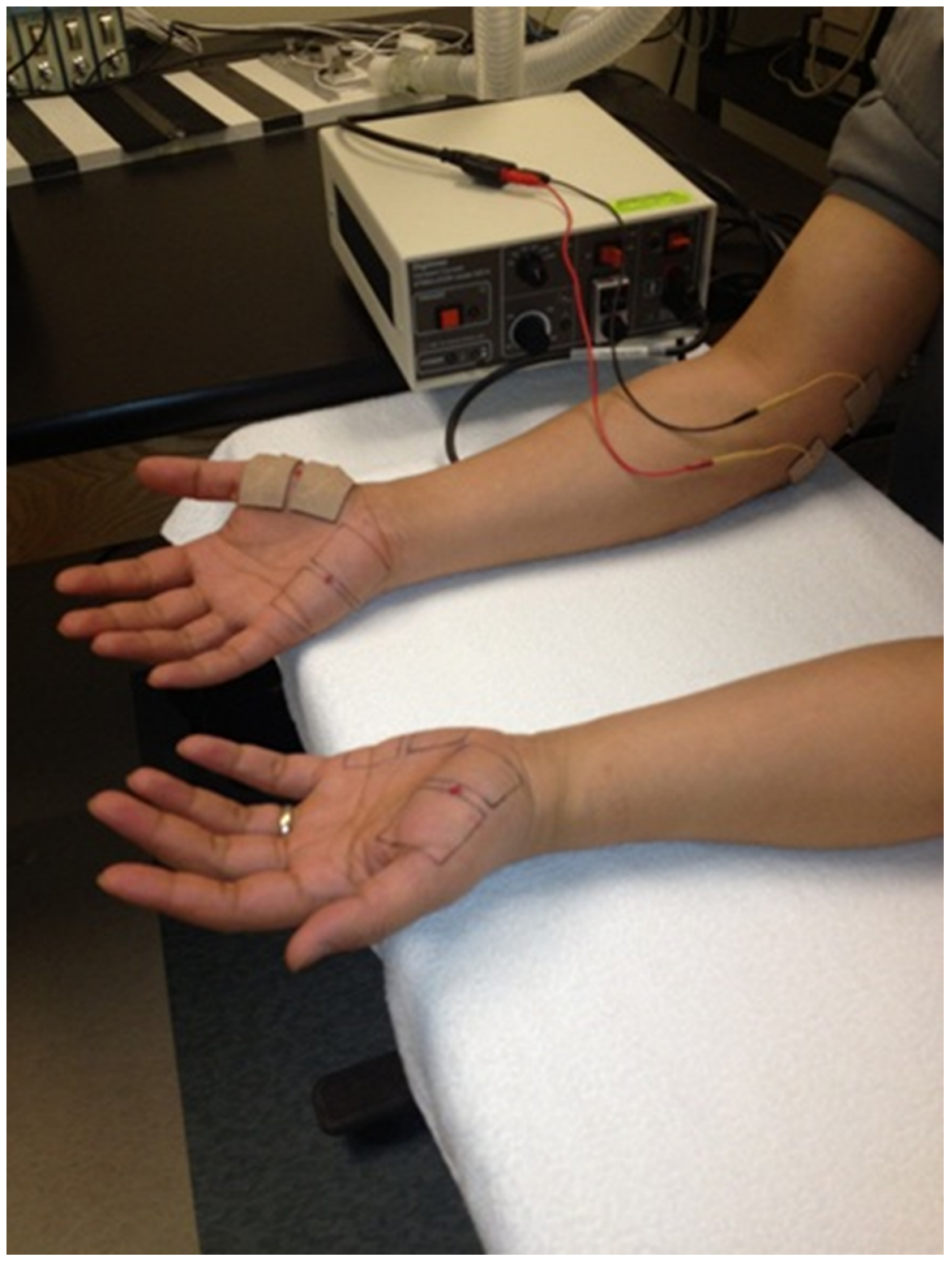
Experimental Set-up.

During each intervention session, subjects were seated comfortably with both arms and hands on the experiment table in approximately symmetrical positions with the elbow joint of the dominant arm flexed at about 90 degrees (Figure 1). A pair of surface electrodes was placed on the medial aspect on the elbow joint. One electrode was at the joint line level and the other was approximately 3 cm above proximally along the path of ulnar nerve. Surface electrodes were trimmed to a size of one inch by one inch prior to placement.

Delivery of electrical stimulation was the key difference between EStim and BreEStim. During EStim, single-pulse electrical stimuli were randomly delivered every 4 to 8 seconds using a computer program. During BreEStim, subjects wore a face mask connected indirectly to the experimental computer via a pneumotach system (Hans Rodulph Inc). A single-pulse electrical stimulus was triggered if a preset threshold was met by voluntary inhalation signals, usually every 4 to 8 seconds. During voluntary inhalation, subjects were instructed to take a deep breath, similar to routine deep breaths, but faster and stronger. To ensure this, subjects were explicitly instructed to expand their chest walls during voluntary, effortful inhalation. Experimentally, the airflow rate was monitored on the computer. When the airflow rate reached 40% of its peak, an electrical pulse was triggered. When wearing a face mask, subjects usually tolerated such breathing well. As in our previous studies [14], [15], [18], no hyperventilation was reported. For both EStim and BreEStim, rest was allowed upon request. Length of rest and number of rest breaks were also upon request.

The intensity of the electrical stimulation started from the pain threshold and increased to the highest level as tolerated. At that level, subjects may find the electrical stimulation annoying, noxious or painful, but still tolerable even if received repetitively. The experimenter(s) verbally encouraged subjects to increase the level of electrical stimulation gradually as tolerated and pointed out that aversion to electrical stimulation was part of the intervention. Subjects were advised that the expected pain level was equivalent to 6∼7 on the 0–10 VAS scale. However, it is important to point out that the intensity of electrical stimulation was adjusted by the subjects themselves according to their subjective feeling of aversiveness. The intensity of electrical stimulation was recorded at the 20th, 40th, 60th, 80th, 100th and 120th trial of each intervention.

### Electrical sensation and pain thresholds

Electrical sensation (EST) and pain (EPT) thresholds were the primary quantitative sensory testing measures. To standardize the tests, EST and EPT testing was performed before and 10 minutes after each intervention (EStim and BreEStim) as in our recent publication [21]. Both EST and EPT were assessed on the thenar and hypothenar eminences of both dominant (treatment) and non-dominant (non-treatment) hands to compare the outcomes of the interventions. The order of EST and EPT assessments on the sites (hypothenar and thenar eminences) was randomized and balanced between two hands.

The same trimmed electrodes were used to assess EST and EPT using the same stimulator (electrical stimulator 7SA, Digitimer). A pair of electrodes was spaced next to each other centered on the thenar or hypothenar eminence. The border of each electrode was marked to ensure consistent placement before and after the intervention. For EST, subjects were instructed to close their eyes and to say “yes” when they explicitly felt electrical stimulation. The intensity of electrical stimulation started from zero and gradually increased in increments of 0.1 mA. For EPT, the intensity of electrical stimulation started from the sensation threshold level and increased in increments of 1 mA. The level of electrical stimulation was recorded as EPT when subjects first reported the electrical stimulation as painful. To improve consistency among subjects, they were advised that the pain threshold level was equivalent to 1 on the 0–10 VAS scale. For both EST and EPT assessments, thresholds from three trials were recorded and averaged.

### Data analysis and statistical analysis

EST and EPT were measured at the thenar and hypothenar eminences on both treated (dominant) and non-treated (non-dominant) hands before and after each intervention. Paired t-tests were used to compare the baseline thresholds prior to BreEStim and EStim treatment on different days. The thresholds were similar across each hand and data were collapsed for the baseline analysis. The pre-treatment baseline value was obtained by averaging across testing sites (thenar and hypothenar) and hands (dominant and non-dominant) for EST and EPT. To assess the effect of each intervention, a repeated measures three-way ANOVA with factors of TREATMENT (2 levels, pre- and post-intervention) and HAND (2 levels, dominant and non-dominant) and SITE (2 levels, thenar and hypothenar) was performed. The effect of each intervention was first calculated using the following equation: percent change  =  (post-intervention – pre-invention)/pre-interventionx100%. To further compare the effects of intervention on EST and EPT between BreEStim and EStim, a repeated-measures three-way ANOVA with factors of INTERVENTION (2 levels, BreEStim and EStim), SITE and HAND was performed. A repeated measures two-way ANOVA was performed with factors INTERVENTION and TRIAL (7 levels, 0, 20, 40, 60, 80, 100, 120) to compare possible differences in the intensity between the two interventions. Post hoc Tukey's HSD tests were performed when there was a significant effect in ANOVA tests. The alpha level required for all statistical significance was set at .05. Data were reported as means in the text and as means ± standard errors in the figures and in the table.

## Results

The pre-intervention baseline EST and EPT values were averaged across sites and hands to compare baseline assessment on different days. EST and EPT are summarized in Table 1. There were no statistically significant differences in these pre-intervention thresholds on different days (paired t-tests, p value: 0.66 for EST and 0.98 for EPT).

**Table 1:** Electrical sensation threshold and electrical pain threshold from the thenar and hypothenar eminences of the treatment (dominant) and non-treatment (non-dominant) hands before and after BreEStim and EStim.

	Elecctrical sensation threshold (EST)				Electrical pain threshold (EPT)			
	Treatment		Non-treatment		Treatment		Non-treatment	
	Thenar	Hypothenar	Thenar	Hypothenar	Thenar	Hypothenar	Thenar	Hypothenar
Pre-BreEStim(mean)	4.12	5.25	4.22	5.20	24.28	27.21	23.99	27.46
*Pre-BreEStim(SE)*	*0.3*	*0.6*	*0.3*	*0.5*	*2.7*	*2.8*	*2.3*	*2.8*
Post-BreEStim(mean)	4.25	5.40	4.09	5.14	**26.72**	**31.70**	**27.20**	**31.30**
*Post-BreEStim(SE)*	*0.3*	*0.6*	*0.3*	*0.4*	*2.5*	*3.1*	*2.6*	*3.1*
Pre-EStim(mean)	4.00	5.32	4.24	5.24	24.16	25.35	23.90	27.43
*Pre-EStim(SE)*	*0.1*	*0.4*	*0.2*	*0.4*	*2.3*	*2.4*	*2.1*	*2.3*
Post-EStim(mean)	3.83	5.40	4.25	4.98	23.65	**28.37**	23.32	26.39
Post-EStim(SE)	0.2	0.5	0.3	0.4	2.9	3.2	2.1	2.9

Bold numbers indicate significant difference from pre-treatment measurements.

**Figure 2:**
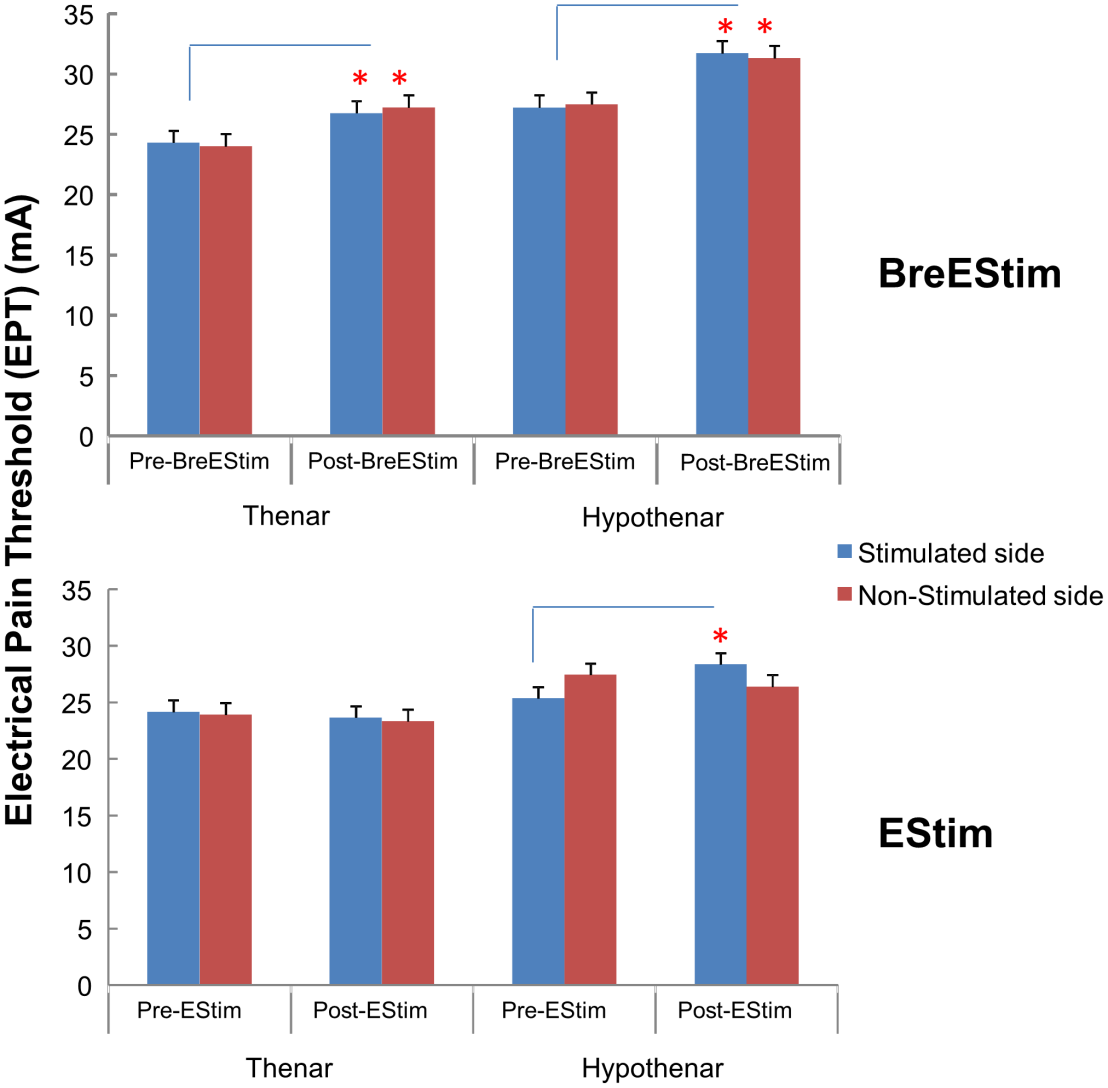
Electrical pain thresholds pre- and post-BreEStim (upper panel) and pre- and post-EStim (lower panel) in all four tested sites on both stimulated and non-stimulated side. Average values and standard errors are shown.

BreEStim systematically increased EPT across sites and hands (Fig 2, upper panel), without changing EST. For EPT, a repeated measures 3-way ANOVA showed main effects of TREATMENT (F_[1, 10]_ = 10.18, p = .009) and SITE (F_[1, 10]_ = 6.98, p = .025). No main effects of HAND or significant interactions were found. EPT averaged across sites and hands significantly increased after BreEStim (Pre vs. Post: 25.7 mA vs. 29.2 mA). EPT was significantly greater in the hypothenar eminence (29.4 mA) than in the thenar eminence (25.5 mA) both before and after BreEStim. A similar ANOVA for EST only showed a main effect of SITE (F_[1, 10]_ = 11.55, p = .007). EST was 4.17 mA in the thenar eminence and 5.25 mA in the hypothenar eminence. No main effects of TREATMENT and HAND, or significant interactions were found.

EStim treatment showed a different effect on EPT, (Figure 2). A 3-way ANOVA did not show main effects of TREATMENT and HAND. But there was a main effect of SITE (F_[1, 10]_ = 6.18, p = .032), significant interactions of TREATMENT x HAND (F_[1, 10]_ = 6.42, p = .030) and TREATMENTxHANDxSITE (F_[1, 10]_ = 8.94, p = .014). Post-hoc Tukey HSD tests revealed that EStim significantly increased EPT on the dominant hypothenar eminence distal to the stimulating site (Pre vs. Post: 25.35 mA vs. 28.37 mA in the dominant hypothenar eminence). EStim did not affect EPT on the contralateral site (Pre vs. Post: 27.43 mA vs. 26.39 mA on the thenar eminence of the non-dominant hand) (see Table 1 for details). Like BreEStim, EStim did not significantly affect EST. A similar 3-way ANOVA only revealed a main effect of SITE (F_[1, 10]_ = 12.27, p = .006) (thenar vs. hypothenar: 4.08 mA vs. 5.23 mA). No other main effects or significant interactions were found.

**Figure 3:**
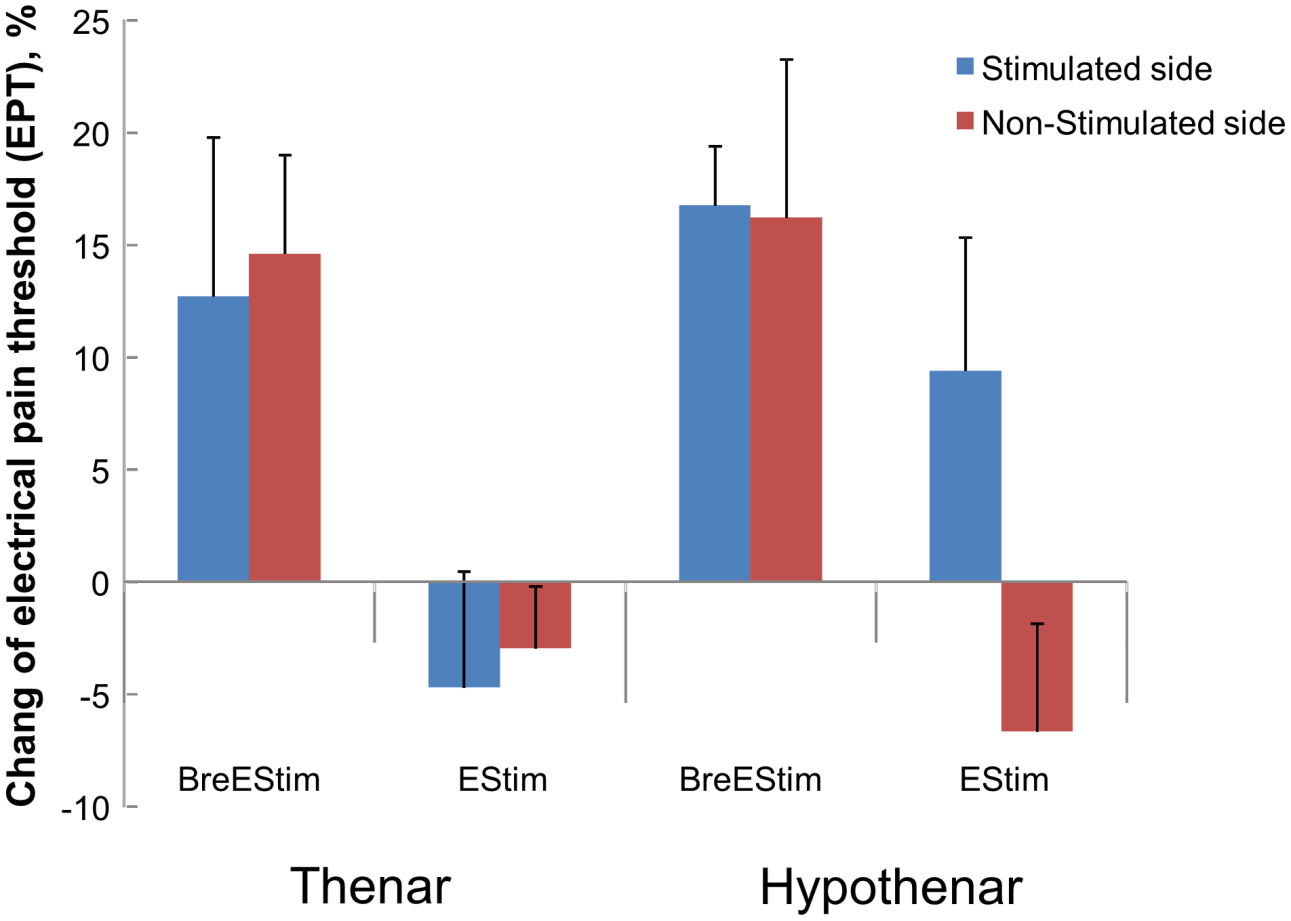
Change of electrical pain threshold as percentage of pre-intervention values after BreEStim and EStim. Average values and standard errors are presented.

Differences in these pre- and post-intervention thresholds reflected the effect of intervention, since the pre-intervention baseline EST and EPT values on different days were not significantly different. EPT change after BreEStim (15.1%) was significantly different from EPT change after EStim (−1.2%) (Figure 3). A 3-way ANOVA showed a main effect of INTERVENTION (F_[1, 10]_ = 9.11, p = .013). No other main effects or significant interactions were found.

**Figure 4:**
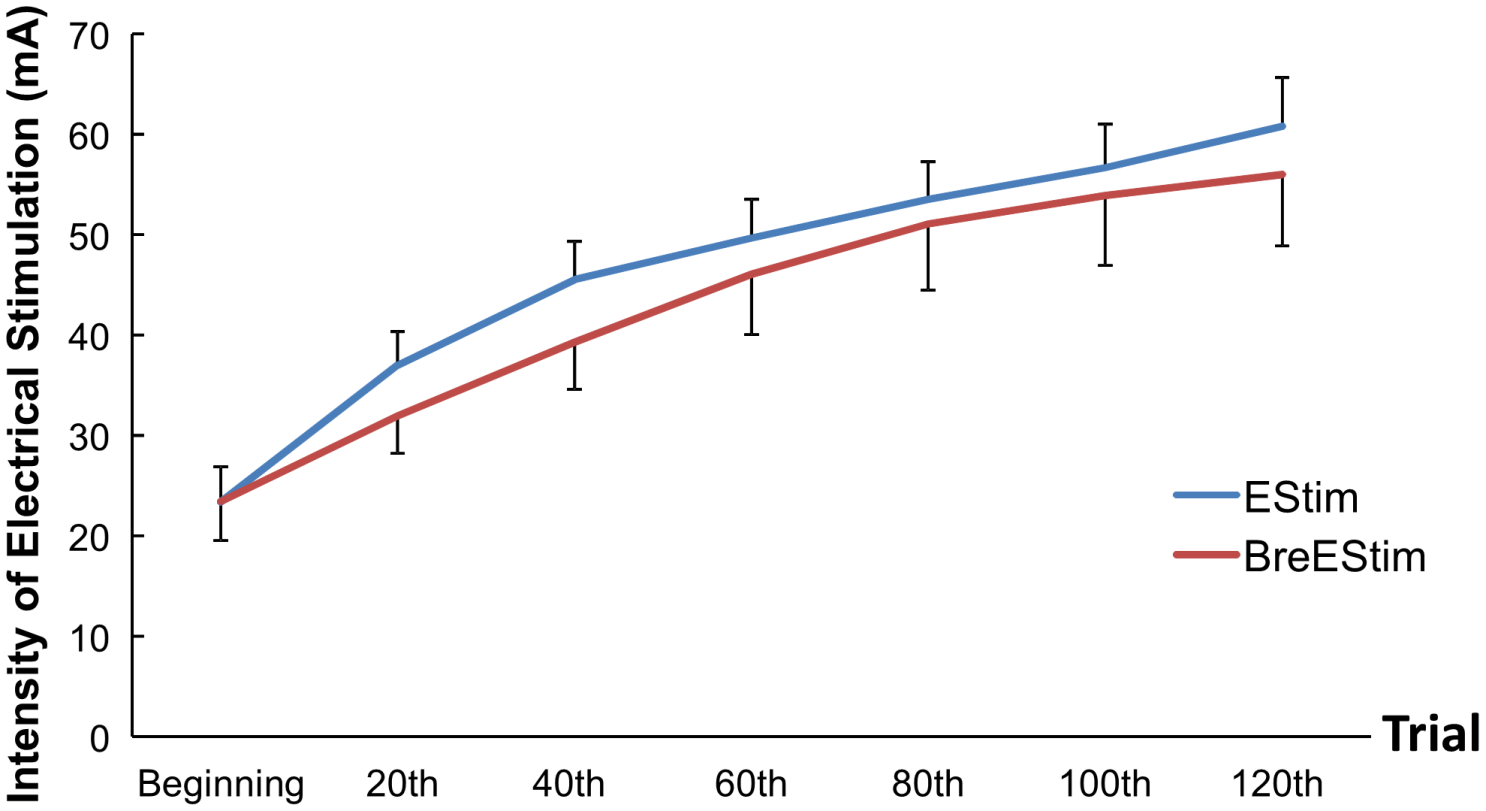
The intensity of electrical stimulation from beginning (trial 0) to the end (trial 120) during BreEStim and EStim. Average values and standard errors are presented.

The intensity of electrical stimulation increased progressively during both EStim and BreEStim (Figure 4). According to an INTERVENTION×TRIAL two-way ANOVA, there was a main effect of TRIAL (F_[6, 60]_ = 48.28, p<.00001), indicating progressive increase in the intensity during the course of treatment. Tukey HSD Post-hoc tests revealed that the intensity increased significantly at the end of 20 trials and 40 trials compared to the starting intensity at the pain threshold (p<0.001). There was no statistical significance in the intensity after 40 trials. However, there was no difference in the intensity between two interventions. The ANOVA showed no main effect of INTERVENTION or significant interaction.

## Discussion

In the present study, pain-free healthy subjects received the same amount of painful, yet tolerable electrical stimuli (120 stimuli) to the ulnar nerve at the elbow level on the dominant side during EStim and BreEStim interventions at least three days apart. Electrical sensation threshold (EST) and electrical pain threshold (EPT) were measured from both thenar and hypothenar eminences of both hands to compare topographic vs. central effects. The main findings were increased EPT in all sites after BreEStim, i.e., habituation, but no effect on EPT after EStim except for the hypothenar eminence on the stimulated side. There was no significant difference in the intensity of electrical stimulation between EStim and BreEStim. Both interventions had no effect on EST.

Overall, the present findings are consistent with the previous study [21]: BreEStim has systemic analgesic effects to painful stimulation after the intervention, i.e., habituation, while there is no such effect after EStim. In the previous study, painful electrical stimuli were delivered to acupuncture points (Neiguan and Weiguan) of the dominant forearm where the acupuncture point – Neiguan is very close to the path of the median nerve Painful electrical stimuli that was delivered to the ulnar nerve at the elbow level (far from the acupuncture points) resulted in the same pattern of analgesic effect across subjects. Variations in individual responses could account for the degrees of difference between two studies. Increase in EPT was about 27% after BreEStim in the previous study. It was 15% in this study. If acupuncture-induced analgesia contributed to the difference, we would have seen further decrease in EPT after EStim in this study as compared to 10% decrease in EPT in the previous study. However, there was no change in EPT in the contralateral non-stimulated hand after EStim.

### Central sensitization after short-term painful electrical stimulation

Central sensitization after a brief course of painful stimulation is a common phenomenon [24]–[26]. In a recent study [24], about 105 stimuli at the intensity of 5 on the visual analogue scale (“10” – worst pain, “0” – no pain) applied to the volar area of the middle forearm caused increased activation in the classic pain processing areas, including the anterior cingulate cortex (ACC), insular cortex, as compared to the rest state. The ACC and the insula have been reported to selectively process the aversive quality of noxious stimulation [27], [28]. Using advanced fMRI (7 T), Hahn et al. [25] was able to demonstrate additional activation of periaqueductal gray (PAG) which was often not observed using 3T fMRI techniques. PAG is known as a pivot region of the descending pain control system [29]–[31]. These studies suggest that painful peripheral electrical stimulation triggers central responses to aversiveness of painful stimuli and internal descending pain coping mechanisms at the same time, i.e., central sensitization.

Our previous findings are consistent with this central sensitization effect of painful EStim [21]. We observed decreased EPT in the thenar eminence in both hands after 100 painful electrical stimuli to the forearm (Neiguan and Weiguan) on the dominant side. However, in the current study we observed increased EPT in the hypothenar eminence distal to the ulnar nerve on the dominant side, but no change in EPT in the thenar eminence of the dominant hand and in both thenar and hypothenar eminences of the non-dominant hand. No change in EPT in the areas that are not innervated by the ulnar nerve may be attributed to the possible habituation effect, since we used the same protocol but a higher number of painful electrical stimuli (120 electrical stimuli) in the present study. However, the result of increased EPT only in the hypothenar eminence distal to the ulnar nerve at the stimulating site may be caused by a different factor. Direct nerve stimulation to the ulnar nerve at the elbow may interfere with orthodromic impulse propagation of electrical stimulation from the distal hypothenar area — the “busy line” effect [32]. Further study is needed to investigate this dose effect and its relation to central sensitization.

### The habituation effect of BreEStim

The habituation effect of BreEStim in the present study was consistent with our previous study in which electrical stimulation was delivered to acupuncture points [21]. There was no difference in the intensity of electrical stimulation between EStim and BreEStim in the present and previous [21] studies. Therefore, the habituation effect of BreEStim is likely attributable to the effect associated with voluntary breathing. Distinctly different from autonomic breathing, voluntary breathing requires extensive cortical and subcortical activation and suppression of brainstem respiratory center for autonomic breathing [33], [34]. According to brain imaging studies, these respiratory-related areas include the primary motor cortex, the premotor cortex, the supplementary motor area, the primary and secondary somatosensory cortices, the insula, the ACC and amygdala, and the dorsolateral prefrontal cortex [35]–[46].

The habituation effect of BreEStim may be explained by multiple internal pain coping mechanisms. Firstly, voluntary breathing-specific cortical activation is likely to make painful stimulation less unpleasant. There are respiratory specific connections between the insula and the ACC and the activity of pulmonary stretch receptors [47], [48]. Secondary to activation of pulmonary stretch receptors from chest wall expansion during voluntary inhalation, the ACC and the insula are thus specifically activated during voluntary inhalation. The ACC and the insula have been reported to selectively process the aversive quality of noxious stimulation [27], [28], but does not influence sensation of the stimulation [49]. The breathing-associated activation in the ACC and the insula has been related to reduction in pain ratings in certain conditions, such as meditation [50]. Secondly, memory of peripheral electrical stimulation is not consolidated. It has been reported that during activation of the insular cortex by localized micro-stimulation, peripheral aversive stimulation leads to item-specific impairment of aversive memory reconsolidation, i.e., anterograde amnesia [51]. In other words, peripheral painful electrical stimulation is likely to be remembered to a lesser degree during BreEStim. In contrast, memory of aversive electrical stimulation is likely to be consolidated during EStim in which electrical stimulation is delivered during normal breathing. Lastly, the descending pain control mechanism is further enhanced by BreEStim. A recent human study supports the important role of the PAG in regulation of both respiration and pain [27]. Activation of PAG is likely further enhanced during voluntary breathing [52], in addition to painful stimulation-induced reflexive activation to peripheral painful electrical stimulation. Thus, the descending pain control role of PAG is further strengthened. Further neuroimaging studies are needed, however, to substantiate the above mechanisms.

Our findings support the important role voluntary breathing plays in habituation to painful peripheral electrical stimulation during BreEStim. The multi-faceted effects on affective and sensation dimensions of pain could explain the effectiveness of BreEStim on pain reduction in amputation and spinal cord injury patients [14], [15]. This is essentially consistent with previous reports of the effect of regulated breathing on reduction in pain perception [50], [53], [54]. After repetitive exposure to thermal pain pulses, pain intensity and unpleasantness were reduced during slow breathing (half of normal rate) as compared to normal breathing in both healthy subjects and subjects with fibromyalgia syndrome. It is worth mentioning that slow breathing also requires voluntary control of breathing. Future imaging studies may be able to detect different activation patterns between effortful and fast vs. slow voluntary breathing.

### Conclusion

In summary, BreEStim increased EPT in all tested sites in both hands, while EStim increased EPT in the hypothenar eminence distal to the stimulating nerve and has no effect on EPT in other sites not innervated by stimulating nerve in pain-free healthy human subjects.
